# Impact of the structure on the thermal burnout effect induced by microwave pulses of PIN limiter diodes

**DOI:** 10.1038/s41598-022-07326-w

**Published:** 2022-02-25

**Authors:** Jingtao Zhao, Quanyou Chen, Zhidong Chen, Chaoyang Chen, Zhenguo Zhao, Zhong Liu, Gang Zhao

**Affiliations:** 1 Science and Technology On High Power Microwave Laboratory, Institute of Applied Electronics, China Academy of Engineering Physics, Mianyang, 621900 China; 2 Key Laboratory of Science and Technology On Complex Electromagnetic Environment, China Academy of Engineering Physics, Mianyang, 621900 China; 3 Institute of Electronic Engineering of China Academy of Engineering Physics, Mianyang, 621999 China; 4 Software Center for High Performance Numerical Simulation, China Academy of Engineering Physics, Beijing, 100088 China

**Keywords:** Applied physics, Electronics, photonics and device physics

## Abstract

Positive-intrinsic-negative (PIN) limiters are widely used to protect sensitive components from leakage power itself and adjacent high-power injection. Being the core of a PIN limiter, the PIN diode is possible to be burnt out by the external microwave pulses. Here, using a parallel computing program for semiconductor multi-physics effects designed by ourselves, we studied the influence of the thickness of the I layer and the anode diameter of the PIN diode on the maximum temperature change curve of the PIN diode limiter. The damage threshold criterion in the numerical simulation was first studied by comparing experimental results with simulation results. Then, we determined the impact of the structure on the thermal burnout effect induced by microwave pulses of PIN limiter diodes.

## Introduction

In the front-end of a radar system, Positive-intrinsic-negative (PIN) limiter is one of the most important modules to protect the back sensitive devices from leakage power itself and adjacent high-power injection^1–3^. However, with the development of the pulse power technology, the widespread use of radar, the electromagnetic environment faced by radar systems is becoming more and more complicated. External microwave pulses can couple into the electronic systems through the antenna and further damage the PIN limiter^3–5^.

Being the core of a PIN limiter, the PIN diode is a sensitive semiconductor device, which is possible to be burnt out by the injected microwave pulses. The burnout of the PIN diode may lead to the failure of the radio frequency front end or even the entire electronics system^6,7^. Thus, many studies have been carried out for damage effects of the microwave pulse for the PIN limiter. Junction burnout, metallization burnout and thermal second breakdown are indicated to be the main causes of the burnout effect by microwave pulses of the PIN diodes^8–11^. However, few literatures about the impact of the structure, especially the I layer thickness and the anode diameter of the PIN diode, on the thermal burnout effect induced by microwave pulses have been reported.

In this work, using the JEMS-CDS Device, a parallel computing program for semiconductor multi-physics effects, we studied the damage threshold criterion in numerical simulation through comparing experimental results and simulation results. And then, we determined the influence of the structure of the PIN limiting diode on the thermal burnout effect caused by the microwave pulse through simulation.

## Structure of the studied PIN limiter

**Figure 1:**
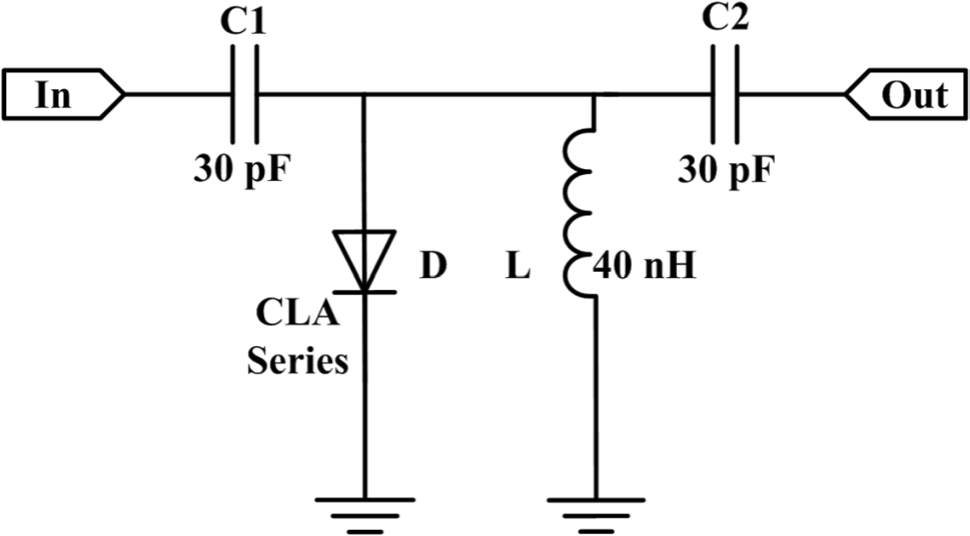
Structure of the single-stage PIN diode limiter used in the study.

**Figure 2:**
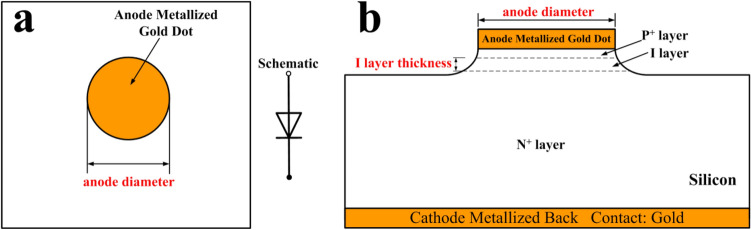
Structure of the model CLA series PIN diode. (**a**) Top view (**b**) Side view.

A typical PIN limiter includes single or multistage PIN diodes. To eliminate the interferences from other factors except the I layer thickness and the anode diameter of the PIN diode, such as other PIN diodes and complex peripheral circuits, a single-stage limiter, whose structure is shown in Fig. 1, is chosen as the target of the study. The typical single PIN diode limiter consists of one PIN diode, two Direct Current (DC) block capacitors, and a parallel inductor. The inductance of the parallel inductor is 40 nH, the DC block capacitors are all 30 pF in this work and the PIN diodes are model CLA series manufactured by Skyworks^12^. The structure of model CLA series PIN diodes whose material is silicon is shown in Fig. 2. The PIN diode mainly consists of a thick substrate and three layers named P^+^, I and N^+^ mounted on it.

## Outline of numerical method and validation

In our numerical methodology, a set of semiconductor equations based on the drift–diffusion model^13^ are at first solved so as to obtain the transient heat source distribution over the PIN diode, The drift–diffusion model includes the following equations.

Poisson equation

1 $$\nabla \cdot \varepsilon_{m} \nabla \varphi = - q\left( {p - n + N_{D} - N_{A} } \right) - \rho_{s}$$

where *ε*_*m*_ is the permittivity of the silicon. *φ* is the electrostatic potential. *q* is the elementary electronic charge. *n* and *p* are the electron and hole density, respectively. *N*_*D*_ and *N*_*A*_ are the density of donors and acceptors, respectively. *ρ*_*s*_ is the fixed charge or interface state charge in the insulating layer.

Continuity equation

2 $$\frac{\partial n}{{\partial t}} = \frac{1}{q}\nabla \cdot {\mathbf{J}}_{{\mathbf{n}}} - \left( {U_{n} - G_{n} } \right)$$

3 $$\frac{\partial p}{{\partial t}} = \frac{ - 1}{q}\nabla \cdot {\mathbf{J}}_{{\mathbf{p}}} - \left( {U_{p} - G_{p} } \right)$$

where **J**_**n**_和**J**_**p**_ are the current densities of electrons and holes, respectively. *G* and *U* are the electron–hole generation and recombination rates, respectively.

Carrier transport equation

4 $${\mathbf{J}}_{{\mathbf{n}}} = qn\mu_{n} {\mathbf{E}}_{{\mathbf{n}}} + k_{b} \mu_{n} \left( {T\nabla n + n\nabla T} \right)$$

5 $${\mathbf{J}}_{{\mathbf{p}}} = qn\mu_{p} {\mathbf{E}}_{{\mathbf{p}}} - k_{b} \mu_{p} \left( {T\nabla p + p\nabla T} \right)$$

where *μ*_*n*_ and *μ*_*p*_ are the mobility of electrons and holes, respectively. *E* represents the intensity of electric field. *T* is the temperature (K). *k*_*b*_ is the boltzmann constant.

When microwave pulses are applied to the PIN diode, the time dependent heat conduction Eq.^14^ will be further solved to get its transient temperature distribution.

6 $$\rho c\frac{\partial T}{{\partial t}} - \nabla \cdot \left[ {\nabla \left( {\kappa T} \right)} \right] = H\left( t \right)$$

where *ρ* is the density (kg/m^3^), *c* is the specific heat (J/kg-K), *κ* is the thermal conductivity (W/m–K), and *H* is the heat generation term (W/m^3^).

The heat generation in the semiconductor is written as

7 $$H\left(t\right)={\varvec{J}}\cdot {\varvec{E}}+\left({E}_{g}+3{k}_{b}T\right)\left(U-G\right)$$

The first term on the right side of the formula is ohmic heating, where ***J*** is the current density vector and ***E*** is the electric field. The second term is the exothermic and endothermic heat caused by the recombination and generation of carriers, where *U* is the carrier recombination rate and *G* is the carrier ionization rate.

Aiming at the research requirements of multi-physical effects mechanism of devices in complex electromagnetic environment, a parallel computing program for semiconductor multi-physics effects, JEMS-CDS-Device, is developed. The program is based on the unstructured grid parallel framework-JAUMIN. It uses the finite volume method (FVM) to discretize and uses the Newton method to get fully coupled solution of the “electric-carrier transport-thermal” problem^15^.

**Figure 3:**
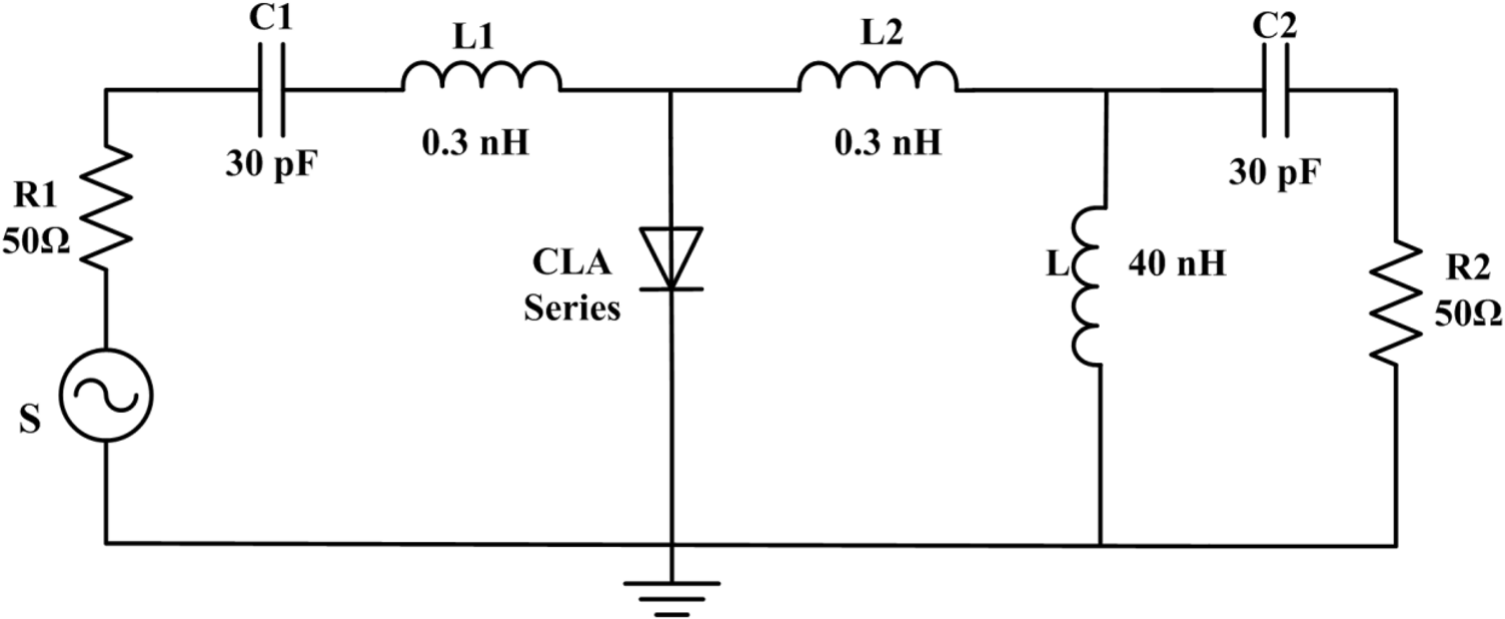
Circuit of the PIN limiter for simulation.

According to the microstrip circuit of the limiter shown in Fig. 1, the simulation circuit of the PIN limiter is established in the simulator as shown in Fig. 3, where S is the microwave pulse source, R1 is the 50 Ω internal resistance of the pulse source, L1 and L2 are the equivalent inductance of the PIN diode welding gold wires, and R2 is the load impedance.

The signal caused by the external electromagnetic pulses coupling into the ribbon cable is similar to be a low-damping sinusoidal voltage signal, which can be approximately expressed as^16^

8 $$U={U}_{0}\left(\mathit{sin}2\pi f+\varphi \right)$$

where *U*_*0*_ is the amplitude of electromagnetic pulses, *f* is the pulse frequency, and *φ* is the initial phase. This simulation does not consider the influence of the initial phase, so the initial phase is set to be 0, the pulse frequency is set to 3 GHz, and the pulse width is set to 100 ns, which are consistent with the experimental settings.

**Figure 4:**
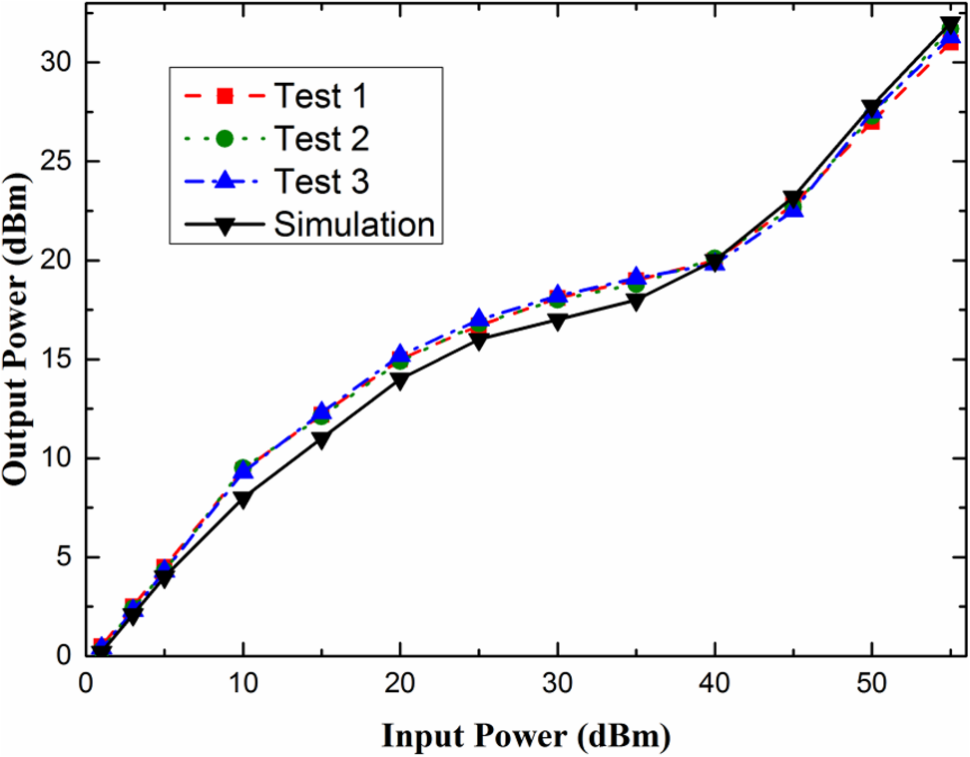
Simulation and test results of typical performance characteristics for the CLA4601 silicon limiters.

**Figure 5:**
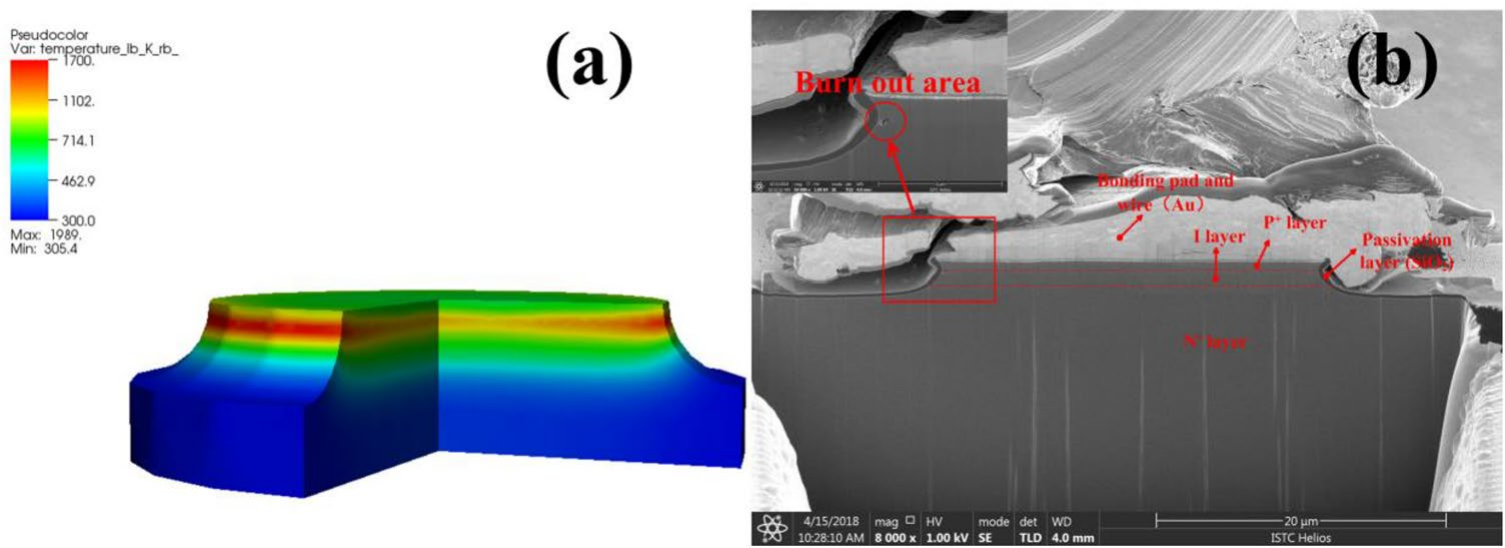
Internal temperature distribution obtained by simulation (**a**) and cross-sectional view via dual beam FIB cross section analysis (**b**) of the burnt-out CLA4601 silicon limiter.

The employed structure parameters were obtained from the data sheet of the CLA series PIN diodes^12^, and the dopant profiles were extracted by semiconductor process simulation. In order to verify the feasibility of the analytical model, take CLA4601 PIN limiter as an example, the typical performance characteristics of the PIN limiters obtained from simulations and experimental measurements were compared and analyzed. As shown in Fig. 4, the simulation are compared with the test data, both are in very good agreement with each other. Figure 5a shows the internal temperature distribution of the burnt-out CLA4601 PIN limiter obtained by simulation. The highest temperature occurs at the junction edge between the P + and I regions of the CLA4601 PIN limiter. Therefore, we speculate that when the device starts to burn out, the first burned position should be at the junction edge between the P+ region and the I region. To further verify the analytical model, the limiter PIN diodes damaged by microwave pulses were physically analyzed via dual beam focused ion beam (FIB) cross section analysis (FEI Helios 600). The cross-sectional view of the limiter PIN diode is shown in Fig. 5b. It can be seen from the Fig. 5 that the burn out area of the device is in perfect agreement with the simulation result. Therefore, the physical models selected for simulation can simulate the physical process of high power microwave injection into the PIN limiters, which can be applied to further preliminary analysis of effect mechanism.

## Numerical results and discussion

The Skyworks CLA series of silicon limiter diodes have two structures, mesa-constructed and planar-constructed. In this study, the widely used mesa structure devices CLA4601, CLA4602, CLA4604 and CLA4605 were selected for effect experiment research. And compared with the simulation results, the device parameters are shown in Table 1.

**Table 1:** Structural parameters of the CLA4601, CLA4602, CLA4604 and CLA4605 silicon limiter diodes.

	CLA4601	CLA4602	CLA4604	CLA4605
H_i_ (μm)	1	1	2	2
L_p_ (μm)	27	29	42	51

**Figure. 6:**
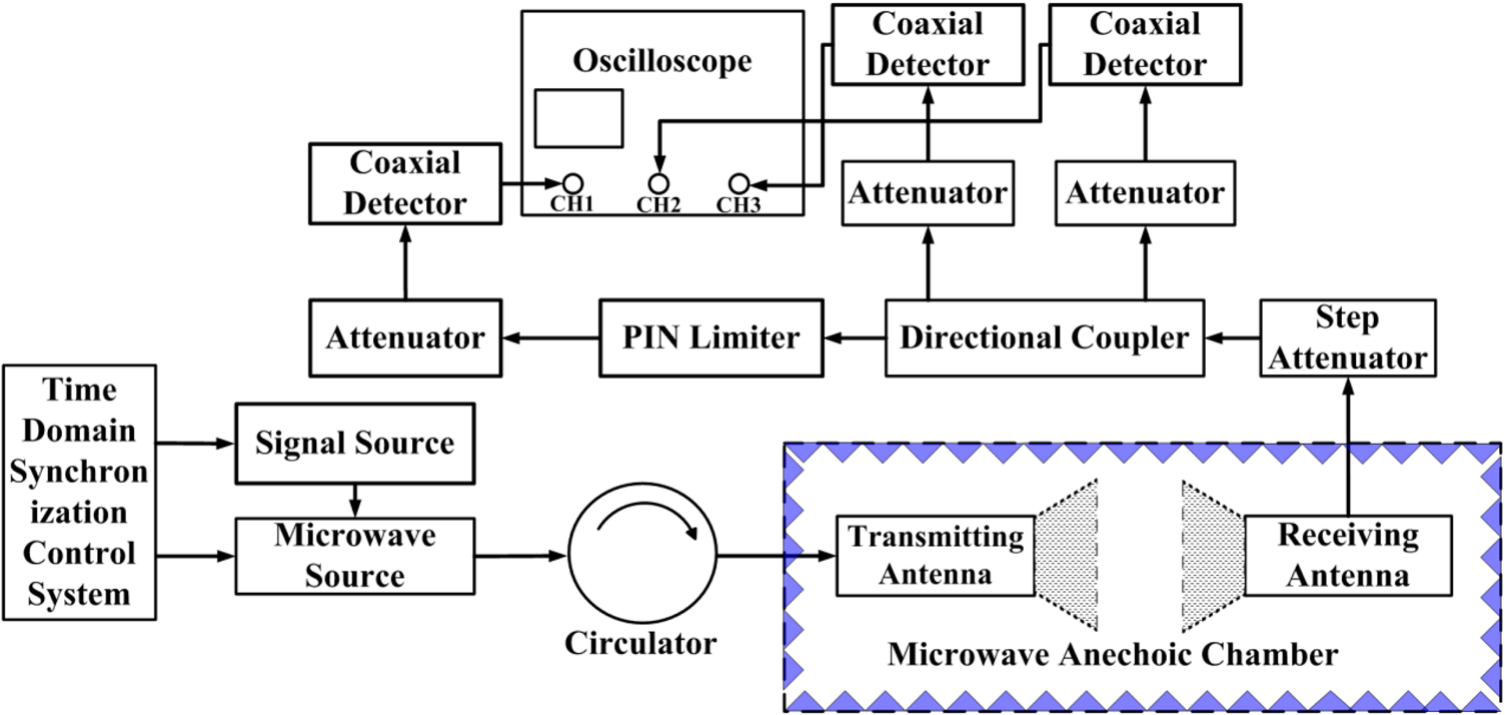
Schematic diagram of the measurement system employed for studying the thermal burnout effect on PIN diode limiters.

In the numerical simulation of the electromagnetic effect of microwave devices, the maximum temperature criterion in a semiconductor device as the melting point of the specific semiconductor material or electrodes is usually used to determine a burnout phenomenon in the simulation^11,17–20^. Therefore, the burnout power thresholds of the PIN limiters are at first simulated based on the peak temperature inside the device reaching the melting point of the material (silicon = 1688 K), as shown in Fig. 6 by red cube.

It is noteworthy that the use of a microwave power limiter generally leads to additional insertion loss in a receiver, which increases its noise figure and reduces its dynamic range^17^. This insertion loss is an important indicator of the microwave power limiters and can be used to evaluate the degree of damage to PIN limiters. In the effect experiment, the limiter insertion loss change of 3 dB was used as the damage criterion.

**Figure 7:**
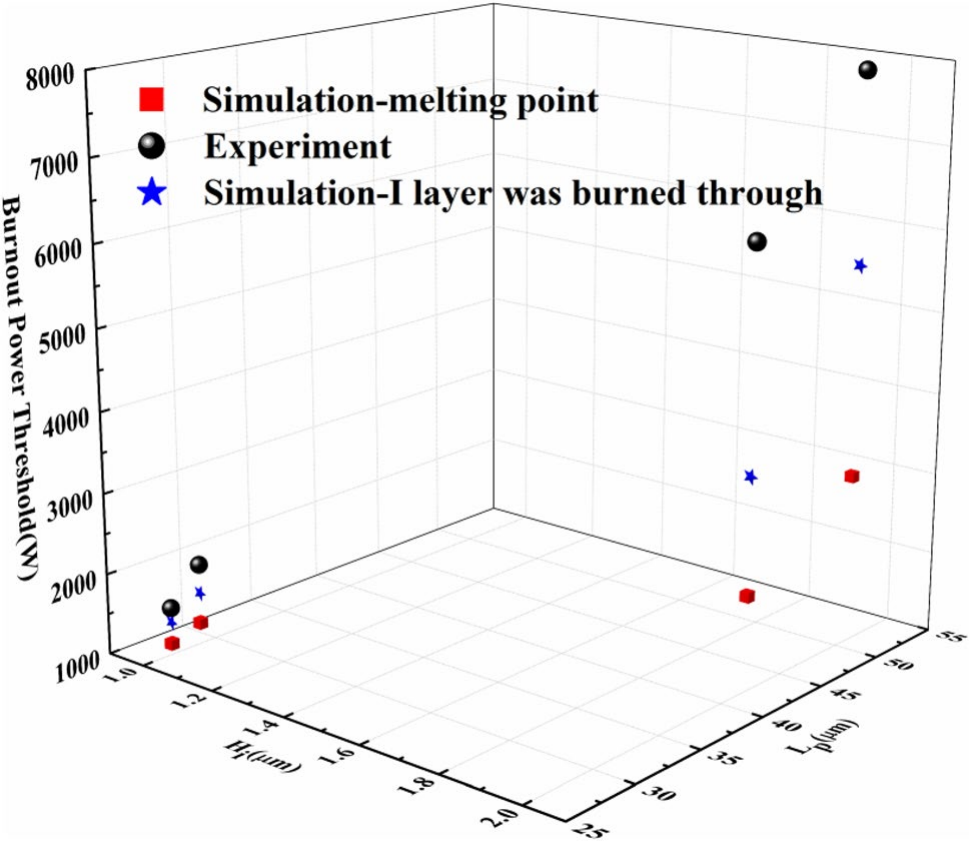
Simulation and experimental results for the CLA4601,CLA4602, CLA4604 and CLA4605 silicon limiters.

Figure 6 shows the schematic of the experimental system employed in our work for studying the thermal burnout effect in PIN diode limiters by injecting microwave pulses into it. This system consists of a self-made microwave source system, several attenuators, directional coupler, coaxial detector (Keysight 8470B), and digital oscilloscope (LeCroy WavePro 640Zi). For our experiments, a series of microwave pulses are generated by the microwave source system, which can be changed gradually by tuning the step attenuator. A self-made time-domain synchronization control system and the signal source (Agilent E8257D) are used to control the pulse width, repetition frequency of the microwave pulses. The conventional microwave pulse parameters (20 Hz repetition frequency and 5 s action time) were selected for the experiments. The results of the device damage threshold obtained by the experiment were shown in Table 2 and Fig. 7 by black ball.

**Table 2:** Simulation and experimental results for the CLA4601, CLA4602, CLA4604 and CLA4605 silicon limiters.

	CLA4601	CLA4602	CLA4604	CLA4605
Experimental results (W)	1585	2000	6310	7943
Simulation-melting point(W)	1139	1264	2171	3080
Simulation-I layer was burned through(W)	1406	1640	3600	5656

It can be seen from Fig. 7 that the experimental results and the simulation results basically have the same trend. The burnout power thresholds increase with the increase of the limiter diodes serial number. However, the experimental thresholds are obviously larger than the simulation results, and the bigger the thickness of the I layer, the more obvious the difference is. When the thickness of the I layer is 1 μm, the experimental result is close to the simulation threshold, and the difference is within 2 dB. When the thickness of the I layer is 2 μm, the experimental result is far from the simulation threshold, and the difference is about 4 dB. The difference between the burnout power thresholds obtained by simulation and experiment are so huge that it cannot meet the practical application.

The above phenomenon may be caused by the inconsistent damage criteria, Preliminary research results^21^ show that it is not accurate to set the maximum temperature criterion in a semiconductor device as the melting point of the specific semiconductor material or electrodes to determine a burnout phenomenon in the simulation. Previous experiments^21^ found that the I layer of the limiter has been basically burned through in the longitudinal direction when the insertion loss changed by 3 dB. Thus, using the hot spot reaching the melting point of the silicon penetrates the I layer as the damage criterion, the burnout power threshold of the limiters were re-simulated. The simulation results were shown by blue star in Fig. 7. It can be seen that using this device damage criterion, the simulation results are closer to the experimental results. When the thickness of the I layer is 1 μm, the difference between the experimental result and the simulation threshold is within 1 dB, and when the thickness of the I layer is 2 μm, the difference between the experimental result and the simulation threshold is about 2 dB. This damage criterion is obviously more reasonable and accurate, and both the trend and the threshold are more consistent with the experimental results.

**Figure 8:**
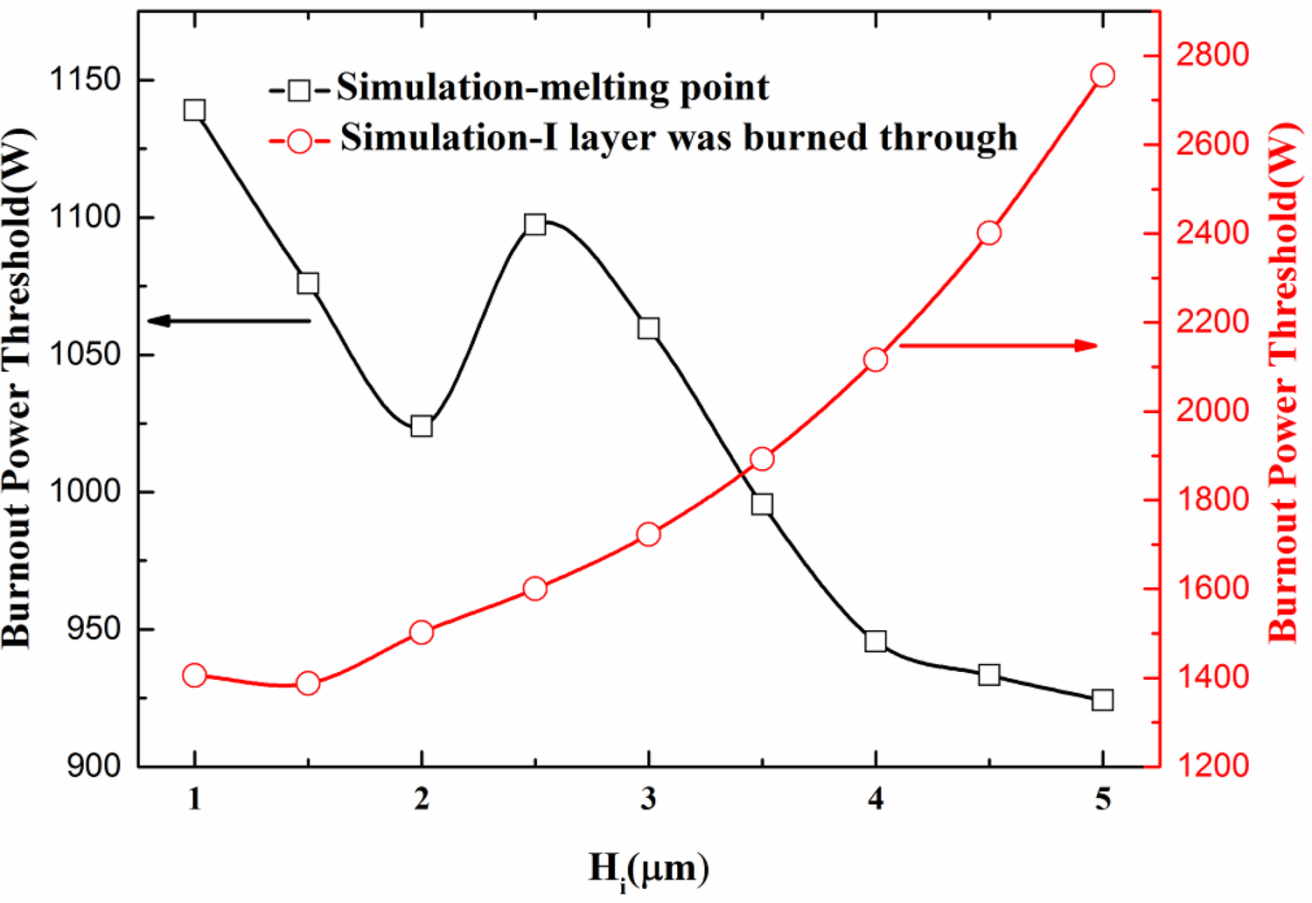
Simulation results of the relationship between the I layer thickness and the burnout power threshold.

In order to study the influence of the I layer thickness on the microwave burnout power threshold of the PIN limiter, the other parameters are the same as those of the CLA4601 PIN diode except for the thickness of the I layer. The damage thresholds of the devices based on the two damage criteria are simulated respectively, and the simulation results of the relationship between the I layer thickness and the burnout power thresholds are shown in Fig. 8.

The simulation results based on the maximum temperature of the device reaching the melting point of the material are shown in Fig. 8 by black square. The burnout power threshold generally decreases as the thickness of the I layer increases. The reason for this phenomenon may be that as the thickness of the I layer increases, the series resistance of the PIN diode increases, and the voltage coupled to the PIN diode die increases accordingly. At the same time, as the thickness of the I layer increases, the charge storage capacity in the I layer increases. So the peak leakage time is longer, that is to say, it need to take longer time to extract the carriers in the I layer to reach the low-resistance limiting state. Therefore, it is more conducive for the PIN diode to absorb more energy to reach the burned state. Also, it should be noted that the burnout power threshold based on the melting point does not change significantly as the thickness of the I layer increases. For example, the difference of the burnout power threshold is just only 0.9 dB between the thickness of the 1 μm and 5 μm I layers.

The simulation results based on the I layer burned through are shown in Fig. 8 by red circle. The burnout power threshold basically increase with the increase of the thickness of the I layer, which is consistent with the usual conclusion. The increase of the I layer thickness will enlarge the thermal power capacity of the PIN diode, so more energy is required to burn the I layer.

**Figure 9:**
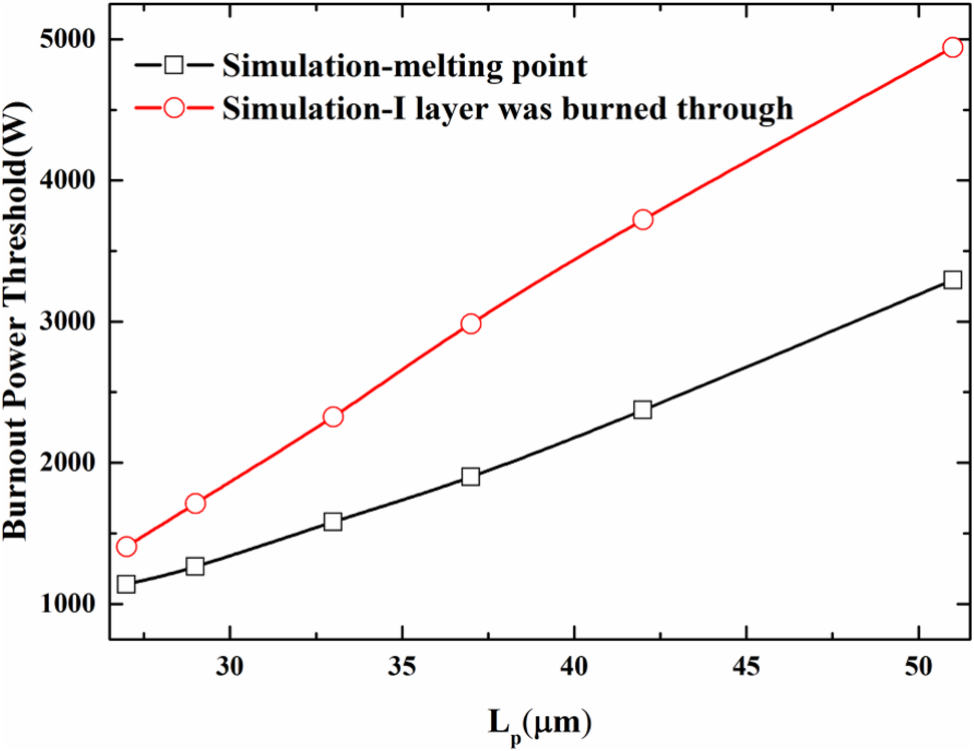
Simulation results of the relationship between the anode diameter of the PIN diode and the burnout power threshold.

Apart from the thickness of the I layer, the anode diameter is also one of the important device parameters of the PIN diode. Although the anode diameter of a specific PIN diode has been determined at the factory, it is also meaningful to study and understand the influence of the anode diameter on the burnout power threshold. In order to study the influence of the anode diameter on the microwave burnout power threshold of the PIN limiter, the other parameters are the same as those of the CLA4601 PIN diode except for the anode diameter. The damage thresholds of the devices based on the two damage criteria are simulated respectively, and the simulation results of the relationship between the anode diameter and the burnout power thresholds are shown in Fig. 9.

It can be seen from Fig. 9 that the anode diameter has a more obvious effect on the burnout power threshold for the burnout of microwave pulse injection. The relationship between the anode diameter of the PIN diode and the burnout power threshold is approximately linear. The main reason for this phenomenon is that the PIN diode with a larger anode diameter has a larger dynamic area (that is, the lateral area of the three layers of P, I and N), which cause the current and thermal power capacity of the device is higher. From the perspective of power density, the larger anode diameter leads to a larger area of the heating disc, and actual received microwave pulse power per unit area is correspondingly lower, which resulting in a higher device burnout power threshold.

## Conclusion

In summary, we investigated the impact of the structure on the thermal burnout effect induced by microwave pulses of the PIN limiter diodes. We found that using the I layer penetrated by the hot spot reaching the melting point of the material as the damage criterion is significantly better than the traditional melting point criterion, both the trend and the threshold are more consistent with the experimental results. This discovery have important reference significance for the analysis of electromagnetic sensitivity of electronic information system and the design of protection and reinforcement of related components.

## References

[1] Cory R (2004). PIN-limiter diodes effectively protect receivers. EDN.

[2] Coaker B (2007). Radar receiver protection technology. Microwave J..

[3] Yi SP, Du ZW (2018). The influence of microwave pulse repetition frequency on the thermal burnout effect of a PIN diode limiting-amplifying system. Microelectron. Reliab..

[4] Backstrom MG, Lovstrand KG (2004). Susceptibility of electronic systems to high-power microwaves: summary of test experience. IEEE Trans. Electromagn. Compat..

[5] Benford, J., Swegle J. A. & Schamiloglu E. Syntax of referencing. In *High Power Microwaves* (2rd ed. Taylor &Francis) 46–53 (New York, 2007).

[6] Leenov D (1964). The silicon PIN diode as a microwave radar protector at megawatt levels. IEEE Trans. Electron Devices.

[7] Tan, R. J., Ward, A. L., Garver, R. V. & Brisker, H. PIN diode limiter spike leakage, recovery time, and damage, *Microw. Symp. Dig.* 275–278 (1988).

[8] Chen X, Du ZW (2010). Effect of pulse repetition frequency on the semiconductor devices burnout caused by microwave pulses. Int. Rev. Electr. Eng..

[9] Wunsch DC, Bell RR (1968). Determination of threshold failure levels of semiconductor diodes and transistors due to pulse voltages. IEEE Trans. Nucl. Sci..

[10] Tasca DM (1970). Pulse power failure modes in semiconductors. IEEE Trans. Nucl. Sci..

[11] Yi SP, Du ZW (2017). The influence of microwave pulse width on the thermal burnout effect of a PIN diode limiting-amplifying system. Microelectron. Reliab..

[12] CLA Series: Silicon Limiter Diode Bondable Chips, Skyworks, Woburn, MA, 2020[Online].Available:https://www.skyworksinc.com/-/media/SkyWorks/Documents/Products/1-100/CLA_Series_200100V.pdf).

[13] Kurata M (1982). Numerical analysis for semiconductor devices.

[14] Synopsys. Taurus Medici-Medici User Guide, Version A-2008.09. 2008.

[15] Li GR (2020). Design and implementation of semiconductor multi-physical parallel computing program JEMS-CDS-Device. High Power Laser Particle Bams..

[16] Korte S, Camp M, Garbe H (2005). Hardware and software simulation of transient pulse impact on integrated circuits. IEEE Trans. Electromagn. Compat..

[17] Yi SP, Du ZW (2019). Thermal burnout effect of a GaAs PHEMT LNA caused by repetitive microwave pulses. IEEE Trans. Plasma Sci..

[18] Yu XH (2015). Analysis of high power microwave induced degradation and damage effects in AlGaAs/InGaAs pHEMTs. Microelectron. Reliab..

[19] Zhou L (2018). Experiments and comparisons of power to failure for SiGe-Based low-noise amplifiers under high-power microwave pulses. IEEE Trans. Electromagn. Compat..

[20] Kuboyama S (2000). Mechanism for single-event burnout of bipolar transistors. IEEE Trans. Nucl. Sci..

[21] Zhao JT (2020). Damage accumulation mechanism in PIN diode limiters induced via multiple microwave pulses. SCI REP-UK.

